# The immune system view of the coronavirus SARS-CoV-2

**DOI:** 10.1186/s13062-020-00283-2

**Published:** 2020-12-29

**Authors:** Ivana Celardo, Luigia Pace, Loredana Cifaldi, Carlo Gaudio, Vincenzo Barnaba

**Affiliations:** 1 Dipartimento di Scienze Cliniche Internistiche, Anestesiologiche e Cardiovascolari, Sapienza Università di Roma, Rome, Italy; 2 Armenise-Harvard Immune Regulation Unit, Italian Institute for Genomic Medicine, FPO IRCCS Candiolo, Turin, Italy; 3 Academic Department of Pediatrics (DPUO),, Ospedale Pediatrico Bambino Gesù,, IRCCS, Rome,, 00165 Italy; 4 Department of Clinical Sciences and Translational Medicine, University of Rome “Tor Vergata”, Via Montpellier 1, Rome, 00133 Italy

**Keywords:** COVID-19, SARS-CoV-2, Vaccine, Coronavirus, Monoclonal antibodies

## Abstract

Knowing the “point of view” of the immune system is essential to understand the characteristic of a pandemic, such as that generated by the Severe Acute Respiratory Syndrome Coronavirus (SARS-CoV)-2, responsible for the Coronavirus Disease (COVID)-19. In this review, we will discuss the general host/pathogen interactions dictating protective immune response or immunopathology, addressing the role of immunity or immunopathology in influencing the clinical infection outcome, and debate the potential immunoprophylactic and immunotherapy strategies required to fight the virus infection.

## Background

Coronaviruses (CoVs) are single-stranded RNA viruses that can cause mild to severe respiratory disease [1]. The coronavirus disease (COVID)-19 due to the Severe Acute Respiratory Syndrome (SARS)-CoV-2 is the third viral severe respiratory disease appearing in these last 20 years, after the two outbreaks by the CoVs belonging to the same betacoronavirus genus, the SARS-CoV which first appeared in November 2002 in Guangdong province (Guangzhou) in China and the Middle East respiratory syndrome (MERS)-CoV, first detected in Jordan and Saudi Arabia in 2012 [2]. Conversely, despite less fatal, SARS-CoV-2 produced a pandemic with a fraction of patients developing severe disease [3, 4]. At the end of December 2019, the Chinese World Health Organization (WHO) office was informed of pneumonia cases with unknown etiology, occurred in the city of Wuhan, in the Chinese province of Hubei. At the beginning of January the reported cases raised to 44, with a quarter (11) displaying severe symptoms. Wuhan was immediately placed under a lockdown regime due to the rapidly growing severity of the infectious health risk. The viral genome was promptly sequenced by Chinese scientists, leading to the identification of a coronavirus similar to the previous SARS-CoV. At the time of writing this review (latest WHO data. Source: Health Emergency Dashboard, October 26, 10.15 am), 42,745,212 are the confirmed cases worldwide since the start of the pandemic, of which 1,150,961 deaths (about 2.7% of infected individuals), particularly among the elderly with various associated pathologies. If we compare these numbers with those of seasonal flu, we find that the latter affects between 5 and 15% of the adult population every year (i.e. from 350 million to 1 billion people), an incidence that rises 20–30% in children, and can evolve into complications that cause death in about 10% of cases, especially among the population groups at risk (children under 5, the elderly and people with chronic diseases). However, in industrialized countries, the flu causes less than 1% of deaths among all the infected individuals, particularly among the elderly with various associated pathologies. This drastic decrease of deaths is particularly due to a more efficient health system in industrialized countries, as well as to the seasonal availability of a vaccine that is protective in more than 40% of individuals. Hence, the fundamental importance of obtaining an anti-SARS-CoV-2 vaccine as soon as possible.

## Immunological diagnostic tests

What measures should be implemented until a vaccine or effective therapies against SARS-CoV-2 will be available? Strict measures to limit damages during the epidemics are in place [5]. These include identification of SARS-CoV-2 RNA in nasal or pharyngeal swabs, and serological tests to identify specific circulating antibodies (both in symptomatic and asymptomatic individuals). Great attention has been dedicated to the serological tests to detect anti-SARS-CoV-2 antibodies. The currently available kits measure a cocktail of antibodies against several viral Spyke (S) determinants, produced in COVID-19 patients after several days from the infection, and do not selectively discriminate the neutralizing antibodies that are capable to inhibit the infection of human cells, by blocking the interaction between the viral S protein and the human angiotensin-converting enzyme 2 receptor (hACE2). The hACE2, a carboxypeptidase that strongly degrades angiotensin II, has been identified as a functional receptor for both SARS-CoV [6] and SARS-CoV-2 [7–9]. Therefore, the positive serology for antibodies tested with the available kits only indicates that a given individual has been exposed to the virus, if the antibodies are of the IgG class, conversely he may even be carrier of the virus, if they were of the IgM class. The serologic tests can be considered an anamnestic marker, and they are of great importance from an epidemiological perspective, as they allow mapping of the infection history of individuals.

The S glycoprotein of SARS-CoV-2 shares 80% amino acid sequence identity with the SARS-CoV S, and includes two functional subunits: S1 (divided into A, B, C and D domains) that is responsible for binding to host cell receptors, and S2 that promotes fusion of the viral and cellular membranes [10]. The identification of neutralizing antibodies recognizing the S domain B (S^B^), the receptor binding domain (RBD) engaging hACE2, is a matter of intense investigation. Recently, it has been identified a human monoclonal antibody (hmAb), among multiple hmAbs targeting SARS-CoV-2 S produced by memory B cells of a SARS survivor infected in 2003, that strongly neutralizes both SARS-CoV-2 and SARS-CoV by engaging the S RBD [11]. This result paves the way for using this hmAb, not only for SARS-CoV-2 prophylaxis or treatment (passive immunization), but also for the identification of serum neutralizing antibodies in functional assays, in which the capacity of neutralizing antibodies (possibly present in convalescent individuals) to inhibit the binding function of the mAb to S RBD is tested. In summary, these promising data suggest that we might be soon able to test the presence of serum neutralizing antibodies and evaluate their long-lasting protective potential.

## Immunity and immunopathology

### Immunity

**Fig. 1:**
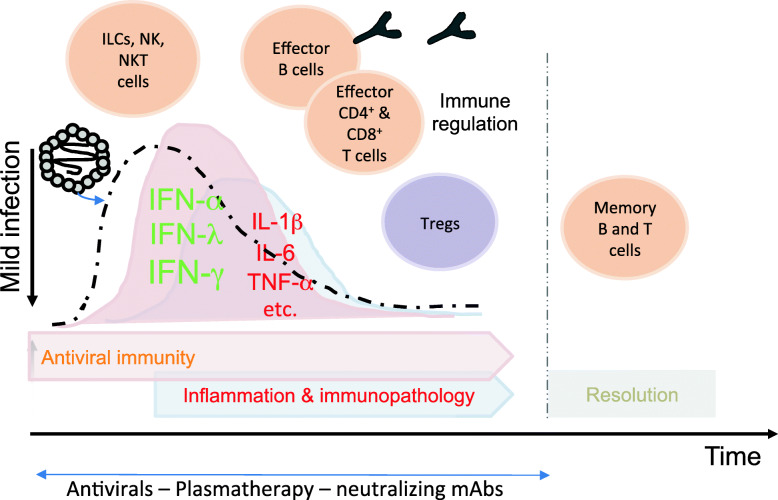
Efficient anti-viral immunity phase as feature of mild infection. Mild infection is characterized by efficient anti-viral immunity phase aimed to eliminate viruses from the host and resolve the infection. A cytokine storm, prevalently formed by anti-viral cytokines (e.g., type-I [IFN-α] and type-III [IFN-λ]) and pro-inflammatory cytokines (IL-6, TNF-α, IL-1-β, etc), is produced by innate immune cells, such as macrophages and DCs. Various innate immune cells (ILCs, NK cells, NKT cells) also intervene to limit viral spread. Consequently, the adaptive responses are mounted to both directly kill virus-infected cells by antigen-specific effector CD8^+^ T cells and to neutralize the virions by antibody producing antigen-specific B cells. IFN-γ production by T cells, as well as by ILCs, NK and NKT cells contribute to viral clearance. Finally, memory T and B cells are generated to guarantee the host protection against secondary infections. An immunoregulatory mechanism mediated by immune checkpoint blockade (e.g., by PD-1, CTLA-4) and Tregs results crucial for the resolution of immunopathology

In order to understand the mechanisms by which the immune system, upon the SARS-CoV-2 recognition, can mount efficient immune responses, it is important to know SARS-CoV-2 tropism and dynamics. The S glycoprotein of SARS-CoV-2 binds hACE2 with significantly higher affinity than SARS-CoV S, and in concert with the host transmembrane serine protease 2 (TMPRSS2) and other host proteases [10, 12], mediates cellular entry. Recently, human, non-human primate, and mouse single-cell RNA-sequencing datasets showed that ACE2 and TMPRSS2 are particularly expressed in lung type II pneumocytes, ileal absorptive enterocytes, nasal goblet secretory cells, and corneal cells [13, 14]**,** supporting the clinical pictures that are more commonly correlated with COVID-19 infection. Importantly, *ACE2* gene was proposed as an interferon-stimulating gene (ISG), suggesting that the resulting production of type I IFNs upregulates ACE2 expression [13]. Tissue-resident macrophages, dendritic cells (DCs), and neutrophils expressing a wide range of pattern-recognition receptors (PRRs), upon the engagement with various damage-associated molecular patterns (DAMPs) and pathogen-associated molecular patterns (PAMPs) (i.e., the single-stranded SARS-CoV-2 RNA binding the endosomal TLRs 7 or 8) [15] induce the activation of distinct signaling pathways promoting production of type I and Type III IFNs [16], as well as of IL-1β and IL-6 promoting recruitment of neutrophils and CD8^+^ T cells, or protective antibody production [17] (Fig. 1). Activated DCs normally patrolling tissues acquire high capacity to phagocytose dying or apoptotic cells (e.g., infected by SARS-CoV-2), upregulate chemokine receptors that guide their migration into draining lymph nodes, and prime virus-specific naive B or T cells to proliferate and differentiate into plasma cells producing anti-viral antibodies and various effector T cell populations, respectively. Effector CD4^+^ and CD8^+^ T cells can then retro-migrate into inflamed tissue to fight the virus through the production of antiviral cytokines suppressing viral replication (e.g., by IFN-γ production), and the antigen-specific killing of infected cells (by CD8^+^ T cells) [18] (Fig. 1). Tissues like respiratory tract or gut contain a large amount of secondary mucosal-associated lymphoid tissues (MALTs) that can contribute to the generation of tissue-resident memory T cells (TRMs) [19–23]. In this context, various natural killer (NK) cell and innate lymphoid cell (ILC) populations expressing a wide repertoire of activating receptors (Rs) and inflammatory cytokines, may play a key role in sustaining tissue inflammation and killing virus-infected cells (upregulating various NKR ligands) in situ [24, 25] (Fig. 1). In addition, MALT-associated invariant NKT cells, γδT cells, or B cell follicles [26–28] need to be taken in consideration in SARS-CoV-2 infection. Whether these immune responses result protective or harmful in COVID-19 infection, it likely depends on whether they are generated in individuals with the genetic and immunological features, more or less reactive to respond promptly or late (Fig. 1). In general, the majority of patients infected with SARS-CoVs develop a multistep cascade leading to efficient immune responses, and ultimately to the recovery. Recent studies showed a high level of SARS-CoV-2-specific CD4^+^ and CD8^+^ T cell activation and expansion in the majority of patients (~ 70–100%) recovering from COVID-19 infection or patients with active infection, consistent with an effective adaptive immune response against several viral epitopes from various proteins (S, M, N, nsps, ORFs…). Interestingly, SARS-CoV-2-reactive CD4^+^ T cells were detected in PBMCs collected several years before the pandemic, in a high percentage of unexposed individuals, suggesting cross-reactive T cell recognition between circulating ‘common cold’ coronaviruses and SARS-CoV-2. Further studied are demanded to ascertain if these responses are effectively protective, correlate with positive outcomes, and provide long-term memory.

Several molecular, cellular, experimental and clinical immunology studies indicate that the majority of effector B or T lymphocytes disappear after pathogen eradication, through the serial intervention of multiple immunoregulatory mechanisms, including those mediated by immune checkpoints (e.g., PD-1 or CTLA-4 interacting with their correspondent ligands [PD-L1 or B7.1 / B7.2] [29], or by Tregs [30, 31], in order to avoid useless damages to the host tissues and organs (Fig. 1). The stop signals provided by immune checkpoints also contribute to develop immunological memory by the conversion of a minority of effector cells into memory B or T cells, through the adaption of several molecular and epigenetic mechanisms [32–37]. Memory lymphocytes remain quiescent in lymphatic tissues or peripheral tissues, but, once they meet again the primary pathogen, they are promptly activated (avoiding the priming period that characterizes a primary infection), neutralize the pathogen, without any disease, and can maintain a lasting and protective immunological memory for several years (long-term memory): this is the principle of vaccinations. The problem is if the immunological memory lasts a few months. Short-term immunological memory occurs when the virus evades the immune responses (e.g., viral persistence), and/or when concurrent causes such as the immune senescence [38] and various co-morbidities (metabolic syndrome, severe generalized immunodeficiencies, tumors, cirrhosis, abuse of alcohol, tobacco or other substances) raise. This leads to recurrent susceptibility to the viral pathogen.

Whether SARS-CoV-2 recalls a long-term immunological memory, similarly to the SARS-CoV (which was however significantly more lethal than SARS-CoV-2), or short term immunological memory, such as other members of the CoVs family, the HCoV-OC43 and HCoV-HKU1 (the second most important causes of common cold), which in winter seasons affects individuals regardless exposures in previous years [3], is a current matter of investigation. The months ahead will be crucial to determine the immunological memory of SARS-CoV-2 by monitoring subjects recovered from a primary COVID-19 [11]. Short-term memory could also convert into long-term memory by the exposure to repeated viral “boosts”, but this remains a pure hypothesis at the moment.

Another important aspect related to the immune response against SARS-CoV-2 is that the CoVs (including SARS-CoV and MERS-CoV) are unique RNA viruses with a genomic proofreading mechanism, that limits the accumulation of mutations [39–41]. This would make these viruses refractory to easy immunosurveillance escape. However, the evidence that the different CoVs frequently recombine their RNA among themselves, suggests that they can undergo a certain degree of variability and capacity of viral escape by this recombination mechanism, in the unluckily scenario that more types of CoVs infect the same cell [40].

### Immunopathology

Whether SARS-CoVs display their pathogenicity through direct cytolytic or indirect non-cytolytic mechanisms, or both, is not completely clarified. The previous SARS-CoV infection in humans caused an atypical pneumonia with a 10% fatality rate, and (in analogy with CoVs of other animals) could induce viral persistence, T cell lymphopenia, and severe disease for several months. This spectrum resembles at least in part the clinical and viral aspects observed in the severe form of COVID-19. The evidence that SARS-CoV-2 can cause different clinical outcomes from asymptomatic to severe symptomatic infection range, leads to hypothesize that it is a poor cytopathic virus, and cell damage is not due to a direct viral effect, but rather by the immune responses elicited to eliminate the virus-infected cells by various effector mechanisms, including killing by CD8^+^ T cells and NK cells, PRR-dependent activation of pro-inflammatory cells (e.g., macrophages, neutrophils…), antiviral and inflammatory cytokines produced by NK cells, NKT cells, ILCs, CD4^+^ and CD8^+^ T cells, TRM cells [42]. Therefore, viral clearance depends on appropriate level of immunopathology that helps production of neutralizing high affinity antibodies by plasma cells [11, 17], and causes recovery in the majority of infected individuals. More in-depth studies on innate and adaptive immunity, during the various phases of the infection, are needed to understand how some patients display an asymptomatic COVID-19, while others a mild or severe symptomatic disease, which can evolve towards the recovery in the majority of them, or the death in a certain fraction. It will be important to understand the checkpoints affected by the virus to overcome the immune system and establish a more or less severe disease that, in the more severe forms, can persist up to more than 2 months.

The diversified clinical outcome of infections is caused by a multifactorial process, to which can contribute and intersect genetic, immune, virus-dependent factors. The most important host genetic factor is represented by the polymorphism of MHC alleles, whose principal function is the presentation of the immunogenic peptides to TCRs on T cells. This MHC capacity likely provides the most reasonable explanation of the relative risk of disease (including autoimmune diseases and infections) in individuals with particular MHC haplotypes [43, 44]. Therefore, it will be critical to study if particular class I and class II alleles are associated with the development of protective immune responses to SARS-CoV-2 or with disease progression (asymptomatic or symptomatic). However, the MHC allele association will represent only a piece of the mosaic that constitutes the multifactorialilty underlying the infection outcome. In addition, genome wide association studies are required to define non-MHC genes associated with COVID-19, including polymorphisms of innate sensor receptors such as NOD-like, or interleukin receptors, despite it is very difficult to define their pathogenic pathways.

**Fig. 2:**
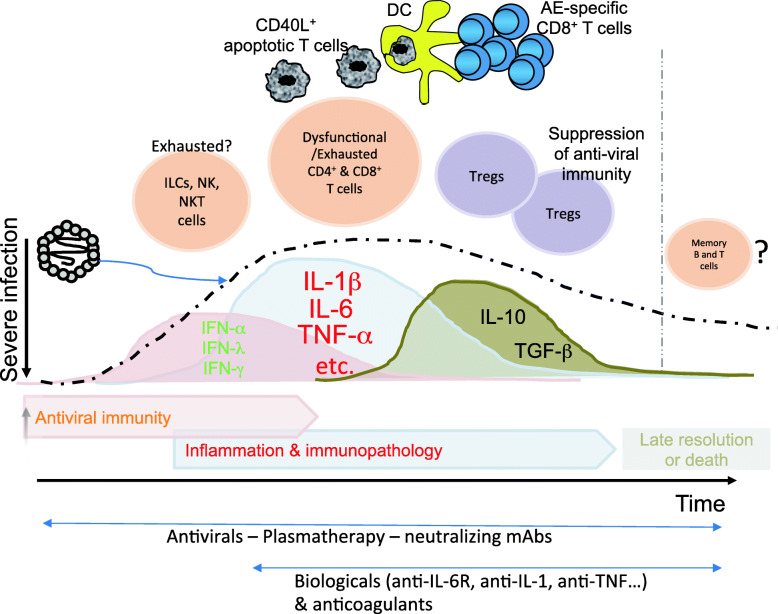
Inefficient anti-viral immunity as feature of severe infection. Severe infection is characterized by inefficient anti-viral immunity and increased immunopathology addressed to provide inflammation (by IL-6, TNF-α, IL-1-β, etc) rather than protection (by IFN-α, IFN-λ, IFN-γ). Effector T cells and likely ILCs and NK cells, which are stimulated by the persisting virus, undergo consecutive steps of exhaustion (partially and then fully exhaustion) and, together with the parallel expansion of Tregs and suppressive cytokines (e.g., IL-10, TGF-β), establish a state of prolonged inflammation. In addition, the hypothesis that BIA is sustained by the expansion of autoreactive CD8^+^ T cells specific to apoptotic epitopes (AEs), which are induced by the cross-presentation of activated apoptotic T cells by DCs, is also considered. Under these conditions, the inefficient anti-viral immunity response does not result in the development of immunological memory. This immune dysregulation leads to severe clinical sequelae (often requiring intensive care units) that undergo restoration in the majority of patients, and death in some of them. The therapeutic approaches will be addressed, firstly, to limit or clear the viral load by various, non-mutually exclusive antiviral strategies (antiviral drugs, plasmatherapy, mAbs neutralizing the virus) in both scenarios displayed in Figs. 1 and 2, to which can be associated various immunotherapy-based biologicals (e.g., anti-IL-6R, anti-IL-1, anti-TNF mAbs), as well as anticoagulants, in an attempt to put out the cytokine storm in the severe form of infection

SARS-CoV-2 may directly antagonize (by their own viral proteins) the first cellular antiviral defense mediated by the transcriptional induction of Type I and III IFN and the subsequent ISGs, as well as demonstrated for SARS-CoV [45]. This hypothesis is consistent with the recent report indicating that the initial host response to SARS-CoV-2 fails to produce efficient type I and type III responses, but induces high levels of a wide array of chemokines recruiting effector cells, including neutrophils as well as adaptive immune cells [46] (Fig. 2). This imbalance would result in the incapacity to promptly stop viral replication, on one hand, and in the maintenance of the inflammatory cascade that is correlated with different levels of disease severity, on other hand. Cytokines, such as IL-1β and IL-6 that are largely secreted by macrophages, as well as a plethora of other inflammatory cytokines including IL-2, IL-8, IL-17, G-CSF, GM-CSF, IP10, MCP1, and TNF are directly correlated with the COVID-19 severity [47] (Fig. 2). This cytokine storm may cause various organ failures including principally the lung and then hearth, liver and kidney, to which contribute the triggering of the coagulatory cascade, generating clots and thrombosis in multiple tissues and organs. The pulmonary impairment is due to the extensive pneumonia, characterized by diffuse alveolar damage with wide infiltration of neutrophils, macrophages, NK cells, activated T cells. The massive compartmentalization of innate and adaptive immune cells in the inflamed tissues may explain the severe peripheral lymphopenia with decreased numbers of CD4^+^ T cells, CD8^+^ T cells, NK cells, and B cells that is correlated with high levels of viral load in severe COVID-19 [47, 48]). NK cells showed lower percentages of CD107a, IFN-γ, IL-2, TNF-α and granzyme B than those in healthy donors in COVID-19 patients [49, 50]. Lung-infiltrating T cells are in particular constituted by terminally effector T cells upregulating different levels of molecules and genes associated with both T cell activation and exhaustion (PD-1, TIM-3, etc) [51] (Fig. 2).

The tissue T cells (likely including the virus-specific) can acquire various functional phenotypes (type-1, type-2, type-17), according to the organ in which they emerge (lung or gut, in particular), and will result protective or detrimental according to disease stage. They may express a partial exhausted phenotype that spontaneously restores into a functional phenotype efficiently limiting or clearing virus replication (recovery) in the mild infection, whereas they progress towards a fully exhausted/dysfunctional phenotype that is generally associated with immunopathology but not with protection, in the severe infection [52, 53]. These two divergent immunological outcomes are epigenetically dictated according to the duration of viral infection and the stimulation strength of virus-specific T cells [54, 55]. Moreover, NK cells were phenotypically exhausted in COVID-19 patients, due to the increased expression of NKG2A [56], an inhibitory receptor able to induce NK cell exhaustion in chronic viral infections [57]. Contextually, further studies are required for ascertaining if the T cell responses against several viral epitopes found in the majority of patients with active infection, result dysfunctional (because exhausted), where they may contribute to maintain the immuno-inflammation, as is the case in chronic (e.g., HBV, HCV) infections [42]. Consistent with this hypothesis, it has been recently proposed that symptomatic COVID-19 behaves more as a subacute rather than an acute disease and may be related with the inability to promptly clear the virus and establish a transient viral persistence [51]. This hypothesis is based on the evidence that SARS-CoV-2 can show a longer median incubation time, a longer disease progression and lymphopenia compared with patients with acute infection, such as influenza [58], and that symptomatic forms of various human SARS-CoV infections can induce viral persistence and T cell lymphopenia.

A further aspect to consider is the constitutive immunological homeostasis regulating the immune responses in frontline organs, such as the respiratory or gut tracts that are continuously exposed to external antigens [19–23]. The maintenance of the local mucosal immunoregulation is mandatory to guarantee the integrity of mucosal tissues and to avoid disastrous chronic inflammations, by limiting immune responses against highly immunogenic microbiota, external pathogens, diet products, or plants. Indeed, these districts are equipped by a very large vascular bed (recruiting neutrophils and memory T cells), and an equally large surface area of MALT (containing B cells producing secretory IgA, macrophages, various types of DCs, intraepithelial T cells, TRMs…) [19, 20, 22, 23]. The mucosal immunoregulation is principally caused by the presence of various types of local Treg subsets suppressing by various mechanisms (CTLA-4–, TGF-β-, IL-10-dependent…), harmful type-1, type-2, type-17, type-22 immune responses that are generated according to innate immune microenvironments of the different districts [32, 59] (Fig. 2). It would be important to investigate the role of Tregs in the various phases of COVID-19 infection. Therefore, the mucosal immunoregulation may consistently contribute to establish a status of prolonged mild-level inflammation in severe COVID-19 infection, in order to avoid excessive tissue damage, on one hand, and the complete suppression of antiviral responses, on the other hand. This scenario is close to the chronic low-level inflammation status occurring in chronic infections, with the main difference that the former is self-limited and runs out when infection finishes, while the latter chronically persists in relation with the viral persistence, for decades and often until death in a remarkable number of patients [42]. This hypothesis is consistent with the observation that the cytokine storm occurring in severe COVID-19 infection is never configured as the so-called “cytokine release syndrome” (CRS) observed in patients with endotoxemia or treated with chimeric antigen receptor-transduced T cells (CAR-T): in these settings, CRS is hyper-acute, shows several fold higher levels of cytokines, neurotoxicity, hypotension and shock, and is significantly more deadly [51].

In this context, a critical phenomenon that may strongly contribute to the intermediate form of CRS observed in severe COVID-19 infection, is the so-called “by-stander immune activation” (BIA), due to (non-virus-specific) T cell responses that contribute to immunopathology during viral infections or various inflammatory diseases [60–63]. BIA can be sustained by several mechanisms [60, 61, 63, 64], including cryptic self-antigens unveiled during the apoptotic T cell turn-over [65, 66] (Fig. 2). Indeed, activated T cells undergoing apoptosis can activate DCs by the interaction between CD40 ligand expressed by (activated) apoptotic T cells and CD40 expressed by DCs [67–69]**.** The so-activated DCs can then phagocytose apoptotic T cells, process caspase-cleaved structural cellular proteins such as myosin, vimentin, and actin, and cross-present the resulting apoptosis-associated epitopes (AEs) to autoreactive CD8^+^ T cells [65]. In turn, the latter undergo apoptosis, upon performing their effector functions, maintaining a vicious circle responsible for the amplification of BIA in various (viral or non-viral) forms of acute or chronic inflammatory diseases [65, 70–73]. Because of the enormous accumulation of activated T cells undergoing apoptosis in the various inflamed districts involved in symptomatic COVID-19 infection, further investigations need to determine if AE-specific T cells may sustain BIA in this infection and correlate with the disease progression (Fig. 2). Among other things, this review can pave the way for setting up novel therapeutic approaches addressed to switch off BIA.

## Vaccines and immunotherapy

The international scientific community is strongly committed to the generation of SARS-CoV-2 vaccine, which can elicit protective immune responses in healthy individuals (active immunization / prophylaxis) (Table 1). Realistically, vaccine against SARS-CoV-2 is probably still months away to be available to everyone, despite the fact that for some of them we will soon have the results of phase III and therefore their effectiveness in terms of protection.

**Table 1:**
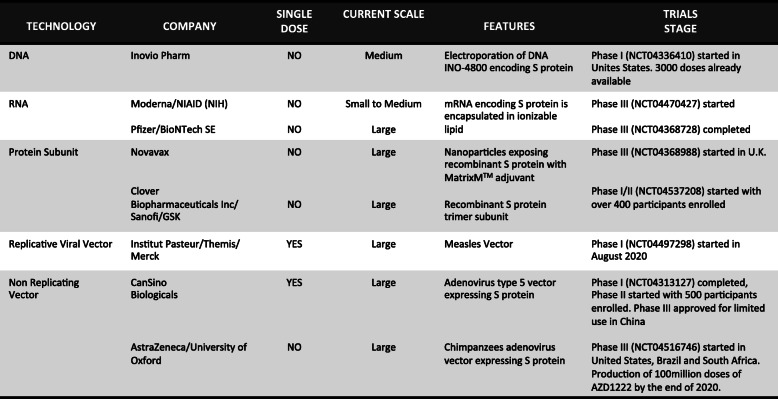
Vaccines development against SARS-CoV-2

If these vaccines, particularly those potentially capable to elicit efficient virus-specific CD8^+^ T cells, can also act as therapeutic vaccines in patients with active infection, is a challenging question, despite the experience with therapeutic vaccines against chronic viral infections are not encouraging. For instance, the soluble HBV protein-based vaccines (the most efficient preventive vaccine: protection from the related infection and hepatocellular carcinoma [HCC] development in more than 95% vaccinated healthy individuals) have been demonstrated to efficiently elicit HBV-specific CD8^+^ T cells, but they do not provide any effect as a therapeutic vaccine in patients with chronic HBV infection or HCC [74, 75]. Because of the various immunosuppressive activities governing the severe viral inflammatory microenvironments, vaccines are likely unable to overcome them, and may need to be used in combination with treatment reagents blocking inhibitory molecules on T cells (e.g., anti-PD-L1, anti-CTLA-4, etc), as the tumor immunotherapy teach us [76–78].

In parallel, the availability of new immunotherapeutic approaches will be essential for the cure of patients. An intriguing approach could be based on the evidence that soluble recombinant (r) fusion protein containing the extracellular domain of hACE2 and the Fc region of the human IgG1 have a high binding affinity for the S RBD of both SARS-CoV and SARS-CoV-2 and neutralize virus pseudotyped with SARS-CoV or SARS-CoV-2 S proteins in vitro [79]. This result proposed to plan clinical trials testing the capacity of the (r)fusion hACE2 protein to neutralize SARS-CoV-2 and prevent severe COVID-19 sequelae (see references in [80]).

A further immunotherapy approach that has been using in various centers is the plasma transfer from individuals recently recovered from COVID-19, into patients with severe disease (plasmatherapy) [81]. The rationale of this approach is based on the possibility that this convalescent plasma may potentially contain neutralizing antibodies, according to ancient technology, called serotherapy. This consists in a transfer of serum from animals immunized with a specific pathogen into infected individuals in order to neutralize the same pathogen or pathogen-deriving toxins (passive immunization). However, the usage of plasma from convalescent COVID-19 patients as a therapy is empiric because it is strictly dependent on the possible presence of high levels of neutralizing antibodies that might be present in convalescent blood donors.

The plasmatherapy could be bypassed by the recent availability of monoclonal antibodies (mAbs) that potently neutralize both SARS-CoV-2 and SARS-CoV by engaging the S RBD [11], which can pave the way for their use in treatment of infected patients, or in passive prophylaxis of healthy individuals, who are frequently exposed to the virus, like hospital staff.

A particular point is the use of the antimalarial drug lysosomotropic agent chloroquine (CQ) in immunotherapy. CQ and its derivatives have been proposed as an antiviral agent in a wide range of infectious diseases, despite it has been never selected as an effective treatment in humans, due its failure in clinical trials [82]**.** To date, no published data or randomized and controlled clinical trials supported the use of CQ in COVID-19 [82–84]. However, a lysosomotropic effect of CQ is that it increases the cross-presentation of soluble antigens to CD8^+^ T cells by professional APCs, principally by enhancing membrane permeabilization at the endosomal level [85]. This effect, together with the ability to inhibit the endosomal acidification and thus the antigen degradation, allows an increased and rapid export of nondegraded antigens from the endosomes into cytosol, favoring thus the class I processing and presentation pathway in vitro and in vivo [85, 86]. Taken together, these data emphasize the usage of CQ as an adjuvant favoring the CD8^+^ T cell responses by prophylactic or therapeutic vaccines rather than a conventional antiviral compound.

During severe COVID-19 infection, the therapies generally used in autoimmune disorders result beneficial to attenuate the clinical sequelae due to the cytokine storm and the BIA (Table 2). For instance, the efficacy of anti-IL-6R mAb observed in various studies to treat patients with severe COVID-19 infection [87], is encouraging the use of mAbs neutralizing other pro-inflammatory cytokines, such as anti-IL-1 [88], anti-IL-17 [89], anti-TNF mAbs [90], or small-molecules inhibiting downstream signalling for blocking cytokine storm-related immunopathology [87]. Accordingly, the beneficial effect of Tocilizumab (anti-IL-6R mAb) treatment in COVID-19 patients has been correlated with the restoration of both T and NK cell number and function [50, 91].

**Table 2:**
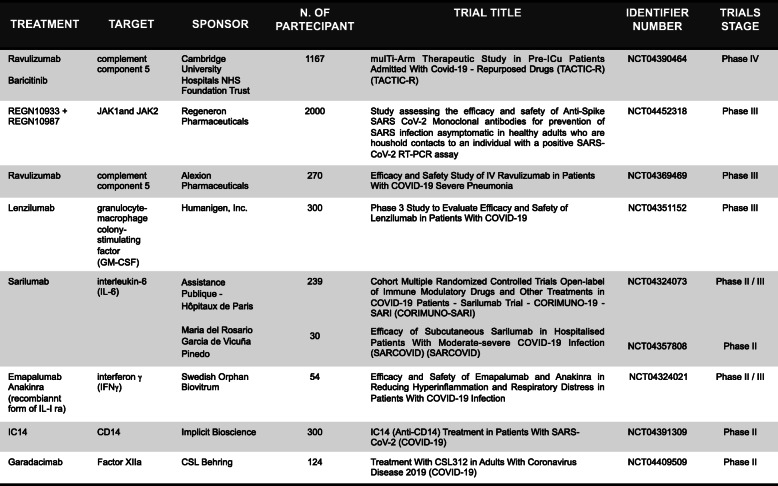
mAbs currently under clinical trials

However, these compounds should be used in association with antiviral drugs or hmAbs neutralizing SARS-CoV-2, in order to avoid the chance to favor wide viral spread if they are used alone (Fig. 1). In addition, the diffuse hypercoagulability leading to multiorgan injury in severe COVID-19 infection, likely due to the combination of the macrophage activation syndrome and direct virus triggering of ACE-2 signaling at endothelial cell level, require various and timely anticoagulant therapeutic approaches [92]**.**

Overall, all the therapeutic strategies listed above require randomized clinical trials to support their potential, and can be beneficial if they are administrated alone or in combination at the right time during the course of the infection (Fig. 1).

## Conclusions

Until the development of an effective vaccine, the results from the clinical trials evaluating the therapeutic alternatives described above are urgently needed. This will be essential for the care of patients, who could increase in number during in the possible following outbreaks, before we have a resolving vaccine.

The immune system taught us how to defend ourselves from the viruses, and the vaccines taught the most extraordinary lesson, since the days of Jerne’s smallpox vaccine (1796). The 1984 Nobel Prize for Medicine Niels Kaj Jerne “poetically” described the immune system as the mirror image of the universe, and the vaccines exploit this amazing capability, in order to elicit the protective immune responses against every small fragment of any pathogen outside of us (“non-self” antigens).

## Data Availability

Not applicable.

## Abbreviations

CAR-T: Chimeric antigen receptor-transduced T cells
CQ: Chloroquine
CoVs: Coronaviruses
DAMPs: Damage-associated molecular patterns
DCs: Dendritic cells
hACE2: Human angiotensin-converting enzyme 2 receptor
hmAb: Human monoclonal antibody
ILC: Innate lymphoid cell
(MERS)-CoV: Middle East respiratory syndrome
MALTs: mucosal-associated lymphoid tissues
NK: Natural killer
PAMPs: Pathogen-associated molecular patterns
PRRs: Pattern-recognition receptors
TMPRSS2: Transmembrane serine protease 2
(SARS-CoV)-2: Severe Acute Respiratory Syndrome Coronavirus
WHO: World Health Organization

## References

[1] Fung TS, Liu DX (2019). Human coronavirus: host-pathogen interaction. Annu Rev Microbiol.

[2] Su S, Wong G, Shi W, Liu J, Lai ACK, Zhou J (2016). Epidemiology, genetic recombination, and pathogenesis of coronaviruses. Trends Microbiol.

[3] Kissler SM, Tedijanto C, Goldstein E, Grad YH, Lipsitch M (2020). Projecting the transmission dynamics of SARS-CoV-2 through the postpandemic period. Science.

[4] Shi Y, Wang Y, Shao C, Huang J, Gan J, Huang X (2020). COVID-19 infection: the perspectives on immune responses. Cell Death Differ.

[5] Cohen MS, Corey L (2020). Combination prevention for COVID-19. Science.

[6] Li W, Moore MJ, Vasilieva N, Sui J, Wong SK, Berne MA (2003). Angiotensin-converting enzyme 2 is a functional receptor for the SARS coronavirus. Nature.

[7] Zhou P, Yang XL, Wang XG, Hu B, Zhang L, Zhang W (2020). A pneumonia outbreak associated with a new coronavirus of probable bat origin. Nature.

[8] Hoffmann M, Kleine-Weber H, Schroeder S, Kruger N, Herrler T, Erichsen S (2020). SARS-CoV-2 cell entry depends on ACE2 and TMPRSS2 and is blocked by a clinically proven protease inhibitor. Cell.

[9] Yan R, Zhang Y, Li Y, Xia L, Guo Y, Zhou Q (2020). Structural basis for the recognition of SARS-CoV-2 by full-length human ACE2. Science.

[10] Walls AC, Tortorici MA, Bosch BJ, Frenz B, Rottier PJM, DiMaio F (2016). Cryo-electron microscopy structure of a coronavirus spike glycoprotein trimer. Nature.

[11] Pinto D, Park YJ, Beltramello M, Walls AC, Tortorici MA, Bianchi S, et al. Cross-neutralization of SARS-CoV-2 by a human monoclonal SARS-CoV antibody. Nature. 2020. 10.1038/s41586-020-2349-y 32422645

[12] Coutard B, Valle C, de Lamballerie X, Canard B, Seidah NG, Decroly E (2020). The spike glycoprotein of the new coronavirus 2019-nCoV contains a furin-like cleavage site absent in CoV of the same clade. Antivir Res.

[13] Ziegler CGK, Allon SJ, Nyquist SK, Mbano IM, Miao VN, Tzouanas CN (2020). SARS-CoV-2 receptor ACE2 is an interferon-stimulated gene in human airway epithelial cells and is detected in specific cell subsets across tissues. Cell.

[14] Sungnak W, Huang N, Becavin C, Berg M, Queen R, Litvinukova M (2020). SARS-CoV-2 entry factors are highly expressed in nasal epithelial cells together with innate immune genes. Nat Med.

[15] Moreno-Eutimio MA, Lopez-Macias C, Pastelin-Palacios R (2020). Bioinformatic analysis and identification of single-stranded RNA sequences recognized by TLR7/8 in the SARS-CoV-2, SARS-CoV, and MERS-CoV genomes. Microbes Infect.

[16] Prokunina-Olsson L, Alphonse N, Dickenson RE, Durbin JE, Glenn JS, Hartmann R (2020). COVID-19 and emerging viral infections: the case for interferon lambda. J Exp Med.

[17] Harker JA, Lewis GM, Mack L, Zuniga EI (2011). Late interleukin-6 escalates T follicular helper cell responses and controls a chronic viral infection. Science.

[18] Piconese S, Timperi E, Pacella I, Schinzari V, Tripodo C, Rossi M (2014). Human OX40 tunes the function of regulatory T cells in tumor and nontumor areas of hepatitis C virus-infected liver tissue. Hepatology.

[19] Rakoff-Nahoum S, Paglino J, Eslami-Varzaneh F, Edberg S, Medzhitov R (2004). Recognition of commensal microflora by toll-like receptors is required for intestinal homeostasis. Cell.

[20] Holt PG, Strickland DH, Wikstrom ME, Jahnsen FL (2008). Regulation of immunological homeostasis in the respiratory tract. Nat Rev Immunol.

[21] Ruddle NH, Akirav EM (2009). Secondary lymphoid organs: responding to genetic and environmental cues in ontogeny and the immune response. J Immunol.

[22] Zhou L, Sonnenberg GF (2018). Essential immunologic orchestrators of intestinal homeostasis. Sci Immunol.

[23] Tokuhara D, Kurashima Y, Kamioka M, Nakayama T, Ernst P, Kiyono H (2019). A comprehensive understanding of the gut mucosal immune system in allergic inflammation. Allergol Int.

[24] Cerboni C, Fionda C, Soriani A, Zingoni A, Doria M, Cippitelli M (2014). The DNA damage response: a common pathway in the regulation of NKG2D and DNAM-1 ligand expression in Normal, infected, and cancer cells. Front Immunol.

[25] Vivier E, Artis D, Colonna M, Diefenbach A, Di Santo JP, Eberl G (2018). Innate lymphoid cells: 10 years on. Cell.

[26] Paget C, Trottein F (2013). Role of type 1 natural killer T cells in pulmonary immunity. Mucosal Immunol.

[27] Hassane M, Demon D, Soulard D, Fontaine J, Keller LE, Patin EC (2017). Neutrophilic NLRP3 inflammasome-dependent IL-1beta secretion regulates the gammadeltaT17 cell response in respiratory bacterial infections. Mucosal Immunol.

[28] Brandtzaeg P, Kiyono H, Pabst R, Russell MW (2008). Terminology: nomenclature of mucosa-associated lymphoid tissue. Mucosal Immunol.

[29] Chen L, Flies DB (2013). Molecular mechanisms of T cell co-stimulation and co-inhibition. Nat Rev Immunol.

[30] Hori S, Nomura T, Sakaguchi S (2003). Control of regulatory T cell development by the transcription factor Foxp3. Science.

[31] Fontenot JD, Gavin MA, Rudensky AY (2003). Foxp3 programs the development and function of CD4+CD25+ regulatory T cells. Nat Immunol.

[32] Sallusto F, Geginat J, Lanzavecchia A (2004). Central memory and effector memory T cell subsets: function, generation, and maintenance. Annu Rev Immunol.

[33] Farber DL, Netea MG, Radbruch A, Rajewsky K, Zinkernagel RM (2016). Immunological memory: lessons from the past and a look to the future. Nat Rev Immunol.

[34] Buchholz VR, Schumacher TN, Busch DH (2016). T cell fate at the single-cell level. Annu Rev Immunol.

[35] Youngblood B, Hale JS, Kissick HT, Ahn E, Xu X, Wieland A (2017). Effector CD8 T cells dedifferentiate into long-lived memory cells. Nature.

[36] Hudson WH, Gensheimer J, Hashimoto M, Wieland A, Valanparambil RM, Li P (2019). Proliferating transitory T cells with an effector-like transcriptional signature emerge from PD-1(+) stem-like CD8(+) T cells during chronic infection. Immunity.

[37] Pace L, Goudot C, Zueva E, Gueguen P, Burgdorf N, Waterfall JJ (2018). The epigenetic control of stemness in CD8(+) T cell fate commitment. Science.

[38] Schober K, Voit F, Grassmann S, Muller TR, Eggert J, Jarosch S (2020). Reverse TCR repertoire evolution toward dominant low-affinity clones during chronic CMV infection. Nat Immunol.

[39] Smith EC, Denison MR (2013). Coronaviruses as DNA wannabes: a new model for the regulation of RNA virus replication fidelity. PLoS Pathog.

[40] Cyranoski D (2020). Profile of a killer: the complex biology powering the coronavirus pandemic. Nature.

[41] Venkatakrishnan AJ, Kayal N, Anand P, Badley AD, Church GM, Soundararajan V (2020). Benchmarking evolutionary tinkering underlying human-viral molecular mimicry shows multiple host pulmonary-arterial peptides mimicked by SARS-CoV-2. Cell Death Dis.

[42] Piconese S, Cammarata I, Barnaba V (2018). Viral hepatitis, inflammation, and cancer: a lesson for autoimmunity. J Autoimmun.

[43] Trowsdale J, Knight JC (2013). Major histocompatibility complex genomics and human disease. Annu Rev Genomics Hum Genet.

[44] Blackwell JM, Jamieson SE, Burgner D (2009). HLA and infectious diseases. Clin Microbiol Rev.

[45] Kopecky-Bromberg SA, Martinez-Sobrido L, Frieman M, Baric RA, Palese P (2007). Severe acute respiratory syndrome coronavirus open reading frame (ORF) 3b, ORF 6, and nucleocapsid proteins function as interferon antagonists. J Virol.

[46] Blanco-Melo D, Nilsson-Payant BE, Liu WC, Uhl S, Hoagland D, Moller R (2020). Imbalanced host response to SARS-CoV-2 drives development of COVID-19. Cell.

[47] Cao X (2020). COVID-19: immunopathology and its implications for therapy. Nat Rev Immunol.

[48] Agrati C, Sacchi A, Bordoni V, Cimini E, Notari S, Grassi G (2020). Expansion of myeloid-derived suppressor cells in patients with severe coronavirus disease (COVID-19). Cell Death Differ.

[49] Zheng M, Song L (2020). Novel antibody epitopes dominate the antigenicity of spike glycoprotein in SARS-CoV-2 compared to SARS-CoV. Cell Mol Immunol.

[50] Mazzoni A, Salvati L, Maggi L, Capone M, Vanni A, Spinicci M (2020). Impaired immune cell cytotoxicity in severe COVID-19 is IL-6 dependent. J Clin Invest.

[51] Vardhana SA, Wolchok JD (2020). The many faces of the anti-COVID immune response. J Exp Med.

[52] Barber DL, Wherry EJ, Masopust D, Zhu B, Allison JP, Sharpe AH (2006). Restoring function in exhausted CD8 T cells during chronic viral infection. Nature.

[53] Wherry EJ, Kurachi M (2015). Molecular and cellular insights into T cell exhaustion. Nat Rev Immunol.

[54] Beltra JC, Manne S, Abdel-Hakeem MS, Kurachi M, Giles JR, Chen Z (2020). Developmental relationships of four exhausted CD8(+) T cell subsets reveals underlying transcriptional and epigenetic landscape control mechanisms. Immunity.

[55] Chu T, Zehn D (2020). Charting the roadmap of T cell exhaustion. Immunity.

[56] Yaqinuddin A, Kashir J (2020). Novel therapeutic targets for SARS-CoV-2-induced acute lung injury: targeting a potential IL-1beta/neutrophil extracellular traps feedback loop. Med Hypotheses.

[57] Li F, Guo Z, Wang H (2013). Influencing elements and treatment strategies associated with the relapse of hepatocellular carcinoma after surgery. Hepatogastroenterology.

[58] Huang C, Peng Y, Zhang Y, Li R, Wan Z, Wang X (2019). Deep dermatophytosis caused by Trichophyton rubrum. Lancet Infect Dis.

[59] Rudensky AY, Campbell DJ (2006). In vivo sites and cellular mechanisms of T reg cell-mediated suppression. J Exp Med.

[60] Lanzavecchia A (1986). Is the T-cell receptor involved in T-cell killing?. Nature.

[61] Tough DF, Borrow P, Sprent J (1996). Induction of bystander T cell proliferation by viruses and type I interferon in vivo. Science.

[62] Ehl S, Klenerman P, Aichele P, Hengartner H, Zinkernagel RM (1997). A functional and kinetic comparison of antiviral effector and memory cytotoxic T lymphocyte populations in vivo and in vitro. Eur J Immunol.

[63] Chu T, Tyznik AJ, Roepke S, Berkley AM, Woodward-Davis A, Pattacini L (2013). Bystander-activated memory CD8 T cells control early pathogen load in an innate-like, NKG2D-dependent manner. Cell Rep.

[64] Ehl S, Hombach J, Aichele P, Hengartner H, Zinkernagel RM (1997). Bystander activation of cytotoxic T cells: studies on the mechanism and evaluation of in vivo significance in a transgenic mouse model. J Exp Med.

[65] Rawson PM, Molette C, Videtta M, Altieri L, Franceschini D, Donato T (2007). Cross-presentation of caspase-cleaved apoptotic self antigens in HIV infection. Nat Med.

[66] Barnaba V (2013). Tuning cross-presentation of apoptotic T cells in immunopathology. Adv Exp Med Biol.

[67] Propato A, Cutrona G, Francavilla V, Ulivi M, Schiaffella E, Landt O (2001). Apoptotic cells overexpress vinculin and induce vinculin-specific cytotoxic T-cell cross-priming. Nat Med.

[68] Griffith TS, Ferguson TA (2011). Cell death in the maintenance and abrogation of tolerance: the five Ws of dying cells. Immunity.

[69] Ivanisenko NV, Seyrek K, Kolchanov NA, Ivanisenko VA, Lavrik IN (2020). The role of death domain proteins in host response upon SARS-CoV-2 infection: modulation of programmed cell death and translational applications. Cell Death Dis.

[70] Franceschini D, Del Porto P, Piconese S, Trella E, Accapezzato D, Paroli M (2012). Polyfunctional type-1, −2, and −17 CD8(+) T cell responses to apoptotic self-antigens correlate with the chronic evolution of hepatitis C virus infection. PLoS Pathog.

[71] Lolli F, Martini H, Citro A, Franceschini D, Portaccio E, Amato MP (2013). Increased CD8+ T cell responses to apoptotic T cell-associated antigens in multiple sclerosis. J Neuroinflammation.

[72] Martini H, Citro A, Martire C, D'Ettorre G, Labbadia G, Accapezzato D (2016). Apoptotic epitope-specific CD8+ T cells and interferon signaling intersect in chronic hepatitis C virus infection. J Infect Dis.

[73] Cammarata I, Martire C, Citro A, Raimondo D, Fruci D, Melaiu O (2019). Counter-regulation of regulatory T cells by autoreactive CD8(+) T cells in rheumatoid arthritis. J Autoimmun.

[74] Barnaba V, Franco A, Alberti A, Benvenuto R, Balsano F (1990). Selective killing of hepatitis B envelope antigen-specific B cells by class I-restricted, exogenous antigen-specific T lymphocytes. Nature.

[75] Boni C, Janssen HLA, Rossi M, Yoon SK, Vecchi A, Barili V (2019). Combined GS-4774 and Tenofovir therapy can improve HBV-specific T-cell responses in patients with chronic hepatitis. Gastroenterology.

[76] Okazaki T, Chikuma S, Iwai Y, Fagarasan S, Honjo T (2013). A rheostat for immune responses: the unique properties of PD-1 and their advantages for clinical application. Nat Immunol.

[77] Sharma P, Allison JP (2015). The future of immune checkpoint therapy. Science.

[78] Rizvi NA, Hellmann MD, Snyder A, Kvistborg P, Makarov V, Havel JJ (2015). Cancer immunology. Mutational landscape determines sensitivity to PD-1 blockade in non-small cell lung cancer. Science.

[79] Lei C, Qian K, Li T, Zhang S, Fu W, Ding M (2020). Neutralization of SARS-CoV-2 spike pseudotyped virus by recombinant ACE2-Ig. Nat Commun.

[80] Amanat F, Krammer F (2020). SARS-CoV-2 vaccines: status report. Immunity.

[81] Duan K, Liu B, Li C, Zhang H, Yu T, Qu J (2020). Reply to Kesici et al. and Zeng et al.: blocking the virus and reducing the inflammatory damage in COVID-19. Proc Natl Acad Sci U S A.

[82] Rebeaud ME, Zores F (2020). SARS-CoV-2 and the use of Chloroquine as an antiviral treatment. Front Med (Lausanne).

[83] Gautret P, Lagier JC, Parola P, Hoang VT, Meddeb L, Mailhe M (2020). Hydroxychloroquine and azithromycin as a treatment of COVID-19: results of an open-label non-randomized clinical trial. Int J Antimicrob Agents.

[84] Boulware DR, Pullen MF, Bangdiwala AS, Pastick KA, Lofgren SM, Okafor EC (2020). A randomized trial of Hydroxychloroquine as Postexposure prophylaxis for Covid-19. N Engl J Med.

[85] Accapezzato D, Visco V, Francavilla V, Molette C, Donato T, Paroli M (2005). Chloroquine enhances human CD8+ T cell responses against soluble antigens in vivo. J Exp Med.

[86] Garulli B, Di Mario G, Sciaraffia E, Accapezzato D, Barnaba V, Castrucci MR (2013). Enhancement of T cell-mediated immune responses to whole inactivated influenza virus by chloroquine treatment in vivo. Vaccine.

[87] Conti P, Gallenga CE, Tete G, Caraffa A, Ronconi G, Younes A (2020). How to reduce the likelihood of coronavirus-19 (CoV-19 or SARS-CoV-2) infection and lung inflammation mediated by IL-1. J Biol Regul Homeost Agents.

[88] Ceribelli A, Motta F, De Santis M, Ansari AA, Ridgway WM, Gershwin ME (2020). Recommendations for coronavirus infection in rheumatic diseases treated with biologic therapy. J Autoimmun.

[89] Pacha O, Sallman MA, Evans SE (2020). COVID-19: a case for inhibiting IL-17?. Nat Rev Immunol.

[90] Feldmann M, Maini RN, Woody JN, Holgate ST, Winter G, Rowland M (2020). Trials of anti-tumour necrosis factor therapy for COVID-19 are urgently needed. Lancet.

[91] Campochiaro C, Della-Torre E, Lanzillotta M, Bozzolo E, Baldissera E, Milani R (2020). Long-term efficacy of maintenance therapy with rituximab for IgG4-related disease. Eur J Intern Med.

[92] Jose RJ, Manuel A (2020). COVID-19 cytokine storm: the interplay between inflammation and coagulation. Lancet Respir Med.

